# From angiography-derived physiology guided PCI to INOCA treatment and to coronary sinus reducer: navigating multiple pathophysiological targets in a single patient: a case report

**DOI:** 10.1093/ehjcr/ytag331

**Published:** 2026-05-14

**Authors:** Jeremie Buri, Adil Salihu, Marion Dupré, Alexandre Berger, Stephane Fournier

**Affiliations:** Department of Cardiology, Lausanne University Hospital (CHUV), Rue du Bugnon 46, Lausanne, Vaud CH-1011, Switzerland; Department of Cardiology, Lausanne University Hospital (CHUV), Rue du Bugnon 46, Lausanne, Vaud CH-1011, Switzerland; Department of Cardiology, Lausanne University Hospital (CHUV), Rue du Bugnon 46, Lausanne, Vaud CH-1011, Switzerland; Department of Cardiology, Lausanne University Hospital (CHUV), Rue du Bugnon 46, Lausanne, Vaud CH-1011, Switzerland; Department of Cardiology, Lausanne University Hospital (CHUV), Rue du Bugnon 46, Lausanne, Vaud CH-1011, Switzerland

**Keywords:** INOCA, Refractory angina, Coronary sinus reducer, Coronary function testing, CFR, IMR, Case report

## Abstract

**Background:**

Intermediate coronary lesions without clear ischaemia should prompt physiological assessment during angiography. Angiography-derived fractional flow reserve enables rapid, wire-free evaluation of lesion significance and can guide revascularization decisions. Persistent angina despite successful percutaneous coronary intervention (PCI) should raise suspicion for concomitant microvascular dysfunction, warranting microcirculation assessment to identify the mechanism and tailor therapy. When no further epicardial target exists and symptoms remain refractory despite comprehensive medical management, coronary sinus reducer (CSR) implantation can be considered. Although these three domains have rapidly evolved over the past few years, they are rarely combined sequentially. This case report illustrates how the stepwise use of these contemporary tools, each addressing a distinct pathophysiological mechanism, led to a marked improvement in the patient’s symptoms and overall clinical course.

**Case summary:**

A 45-year-old man presented with acute coronary syndrome and underwent multivessel PCI in 2023. One year later, he re-presented with unstable angina and received optical coherence tomography-guided left main to left anterior descending artery stenting. However, symptoms persisted (Canadian Cardiovascular Society class II). Invasive coronary function testing revealed impaired vasodilatory reserve with preserved microvascular resistance and no inducible spasm, suggesting functional impairment of vasodilatory capacity. Mechanism-matched therapy with calcium-channel blockade and angiotensin-converting enzyme inhibition provided partial relief. Refractory angina ultimately led to CSR implantation with complete resolution of symptoms.

**Discussion:**

This case highlights a stepwise physiology-first approach, progressing from epicardial revascularization to ischaemia with no obstructive coronary arteries phenotyping and ultimately venous outflow modulation for refractory angina.

Learning points

- Persistent or recurrent angina after apparently successful PCI should prompt invasive coronary function testing to identify microvascular or vasospastic mechanisms.
- Reduced coronary flow reserve with preserved IMR suggests impaired vasodilatory reserve and may indicate functional coronary microvascular dysfunction.
- In selected patients with refractory angina and no further revascularization targets, Coronary Sinus Reducer implantation may represent a therapeutic option.

## Introduction

Intermediate lesions without clear evidence of ischaemia warrant physiological assessment. Angiography-derived fractional flow reserve (FFRangio) has emerged as a potential tool for physiological lesion assessment, with several studies demonstrating good agreement with invasive pressure-wire measurements in selected clinical settings.^1,2^ Nevertheless, pressure-wire fractional flow reserve (FFR) remains the reference standard for invasive physiological assessment. Its accuracy, clinical feasibility, inter-operator reproducibility and impact on revascularization strategy have been validated across the spectrum of acute coronary syndromes, including ST-elevation myocardial infarction in recent dedicated studies.^2–4^

By providing reliable functional information directly from standard angiography, FFRangio supports physiology-guided percutaneous coronary intervention (PCI) and complements the evidence established by invasive FFR trials demonstrating improved outcomes over angiography-guided strategies.^5^ This approach helps optimize lesion selection and avoid unnecessary stent implantation.

When revascularization is anatomically and functionally successful but angina persists, invasive coronary function testing, including calculation of coronary flow reserve (CFR), index of microcirculatory resistance (IMR), and acetylcholine provocation, helps identify the dominant mechanism and tailor therapy accordingly.^6^

In patients without further epicardial targets and persistent symptoms despite guideline-directed medical therapy, treatment options are limited. For carefully selected cases driven by non-epicardial mechanisms, coronary sinus reducer (CSR) implantation has emerged as a mechanism-directed intervention for refractory angina.^7^

This case illustrates a stepwise physiology-first strategy, evolving from angiography-guided PCI to invasive microvascular phenotyping and ultimately venous outflow modulation for refractory angina (Summary figure).

## Summary figure

Timeline of clinical events and mechanism-targeted management in this post-PCI angina case.

**Figure:**
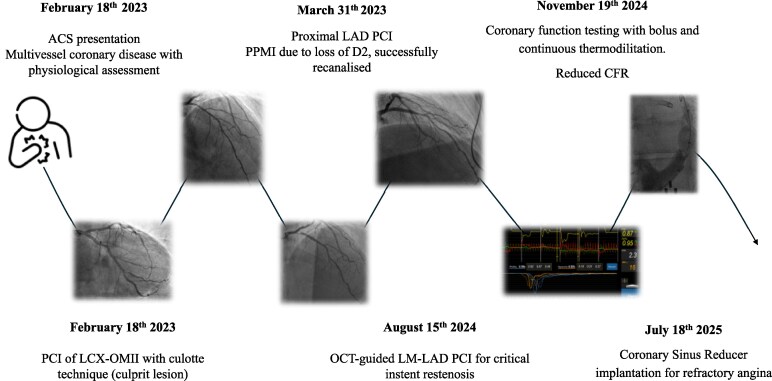
Sequential timeline of the patient’s clinical course. The case evolved from an initial ACS treated with bifurcation PCI (LCx-OM2, February 2023) and proximal LAD PCI complicated by peri-procedural MI (March 2023), through optical coherence tomography (OCT)-guided left main to left anterior descending artery (LM–LAD) PCI for type 4b MI (August 2024), to invasive coronary function testing showing reduced CFR without spasm (November 2024), and finally off-label CSR implantation for refractory angina (July 2025). Abbreviations: ACS, acute coronary syndrome; CFR, coronary flow reserve; CSR, coronary sinus reducer; LAD, left anterior descending artery; LCx, left circumflex artery; LM, left main; OCT, optical coherence tomography; OM2, second obtuse marginal branch; PCI, percutaneous coronary intervention; PPMI, peri-procedural myocardial infarction.

## Case presentation

**Figure 1:**
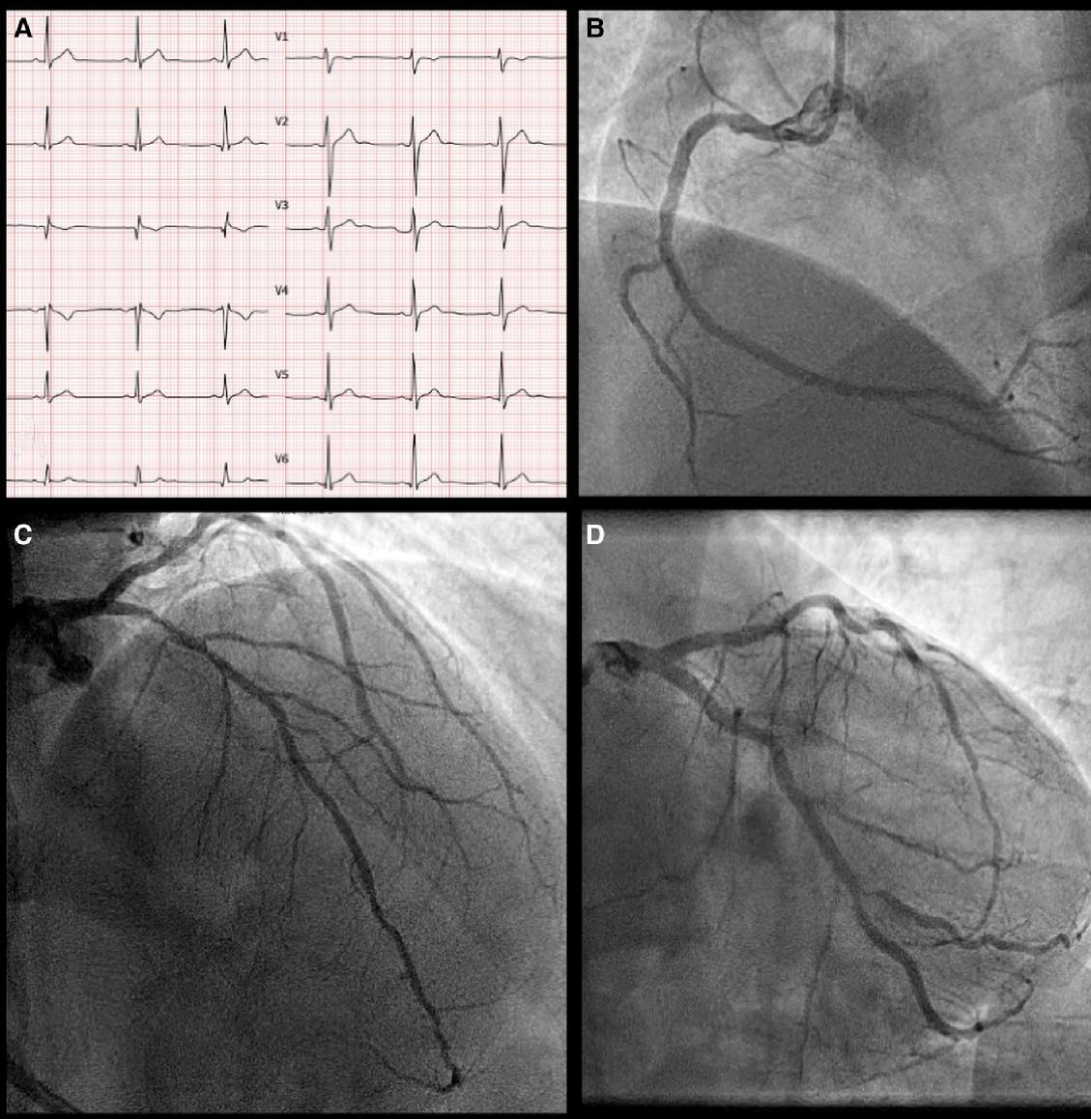
Baseline electrocardiogram and diagnostic coronary angiography at presentation. (*A*) Twelve-lead ECG at admission showing sinus rhythm with isolated T-wave inversion in lead III and minor non-specific repolarization changes, without acute ischaemic ST-segment deviation. (*B*) Right coronary angiography in left anterior oblique projection showing a dominant RCA with mild mid-segment stenosis (<30%) and normal distal flow. (*C*) Left coronary angiography in right anterior oblique caudal (spider) view demonstrating severe bifurcation disease involving the mid-LCx and -OM2 (70%–90%, type B1) and a functionally significant proximal LAD stenosis (FFR = 0.77; LCx FFR = 0.74), corresponding to the culprit lesion. (*D*) Left coronary angiography in left anterior oblique cranial projection confirming significant proximal LAD disease extending to the first diagonal branch, with preserved distal perfusion. Abbreviations: ECG, electrocardiogram; FFR, fractional flow reserve; LAD, left anterior descending artery; LCx, left circumflex artery; OM2, second obtuse marginal branch; PCI, percutaneous coronary intervention; RCA, right coronary artery.

A 45-year-old man with hypercholesterolaemia, a history of active tobacco use and a family history of pre-mature coronary artery disease presented in February 2023 with unstable angina. Physical examination was unremarkable, with stable haemodynamics and no signs of heart failure. Coronary angiography demonstrated multivessel disease with an ostial culprit second obtuse marginal branch (OM2) bifurcation lesion (Medina 00,1), pathological by FFRangio (*Figure 1*).

**Figure 2:**
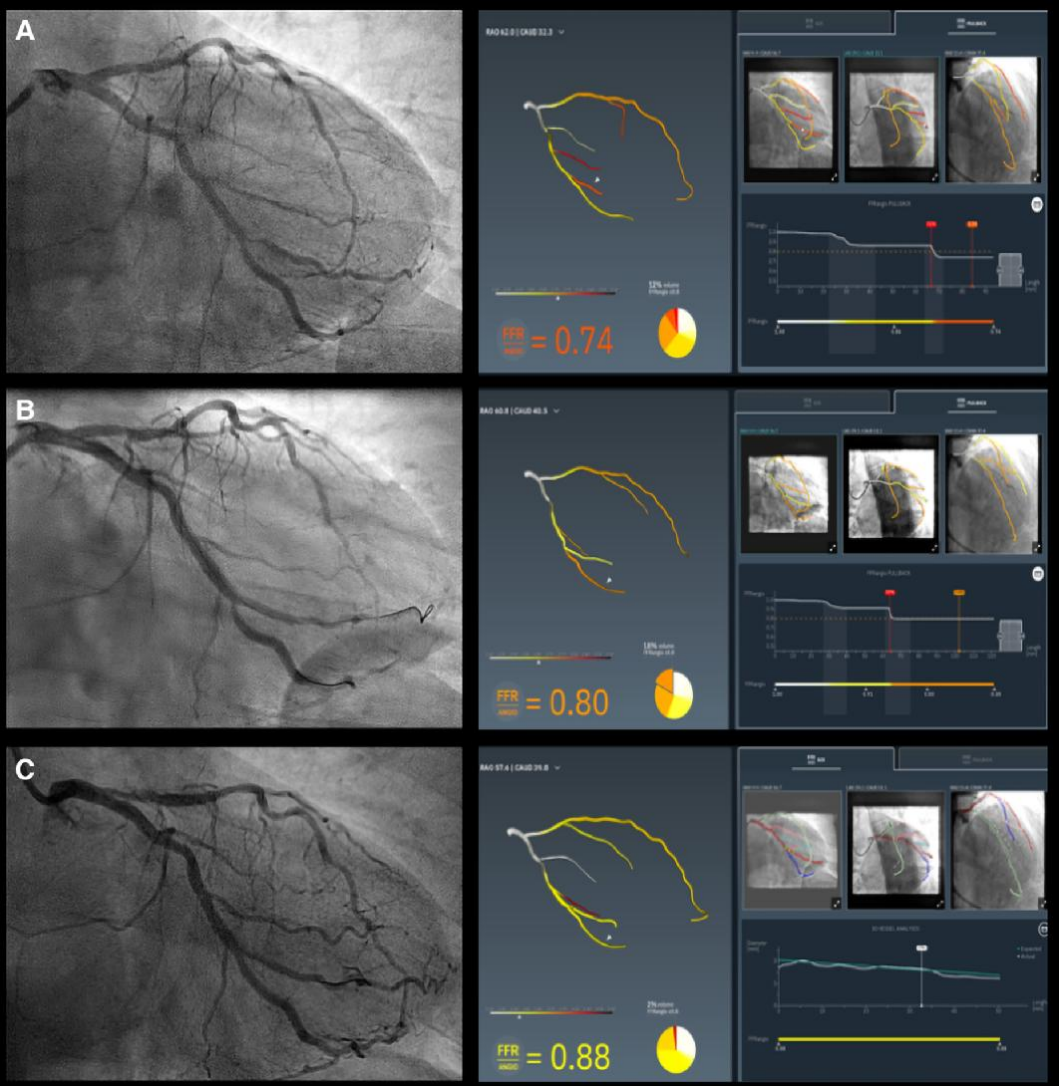
Angiography-derived FFR before and after PCI of the LCx-OM2 bifurcation. (*A*) Pre-PCI FFRangio showing functionally significant flow limitation in the mid-LCx and -OM2 (FFR = 0.74). (*B*) Immediate post-PCI FFRangio after bifurcation stenting showing physiological improvement (FFR = 0.80). (*C*) Final optimization result with distal vessel analysis confirming restoration of physiological flow (FFR = 0.88). Abbreviations: FFR, fractional flow reserve; LCx, left circumflex artery; OM2, second obtuse marginal branch; PCI, percutaneous coronary intervention.

PCI was performed [from the left circumflex artery (LCx) to the OM2] using a provisional strategy later converted to a culotte technique due to a plaque shift in the distal LCx, also pathological by FFRangio, with post-PCI FFRangio confirming a satisfactory physiological result (FFR 0.88) (*Figure 2*). A functionally significant proximal LAD lesion (FFR 0.77) was scheduled for staged intervention. Initial medical therapy included aspirin 100 mg once daily, ticagrelor 90 mg twice daily, metoprolol succinate 37.5 mg once daily, high-intensity lipid-lowering therapy (atorvastatin 80 mg, ezetimibe 10 mg, alirocumab 75 mg every 2 weeks), and pantoprazole 40 mg once daily.

In March 2023, staged PCI of the proximal LAD was performed after intravascular lithotripsy, with implantation of overlapping drug-eluting stents. A peri-procedural myocardial infarction due to loss of the second diagonal branch (D2) was promptly recanalized with restoration of thrombolysis in myocardial infarction 3 flow. The patient recovered uneventfully with a left ventricular ejection fraction (LVEF) of 62%.

Despite optimal medical therapy, he re-presented in August 2024 with exertional angina. Repeat angiography demonstrated a very tight stenosis at the proximal ostium of the LAD stent treated by OCT-guided LM–LAD PCI. Ranolazine was added to optimize anti-anginal therapy.

**Figure 3:**
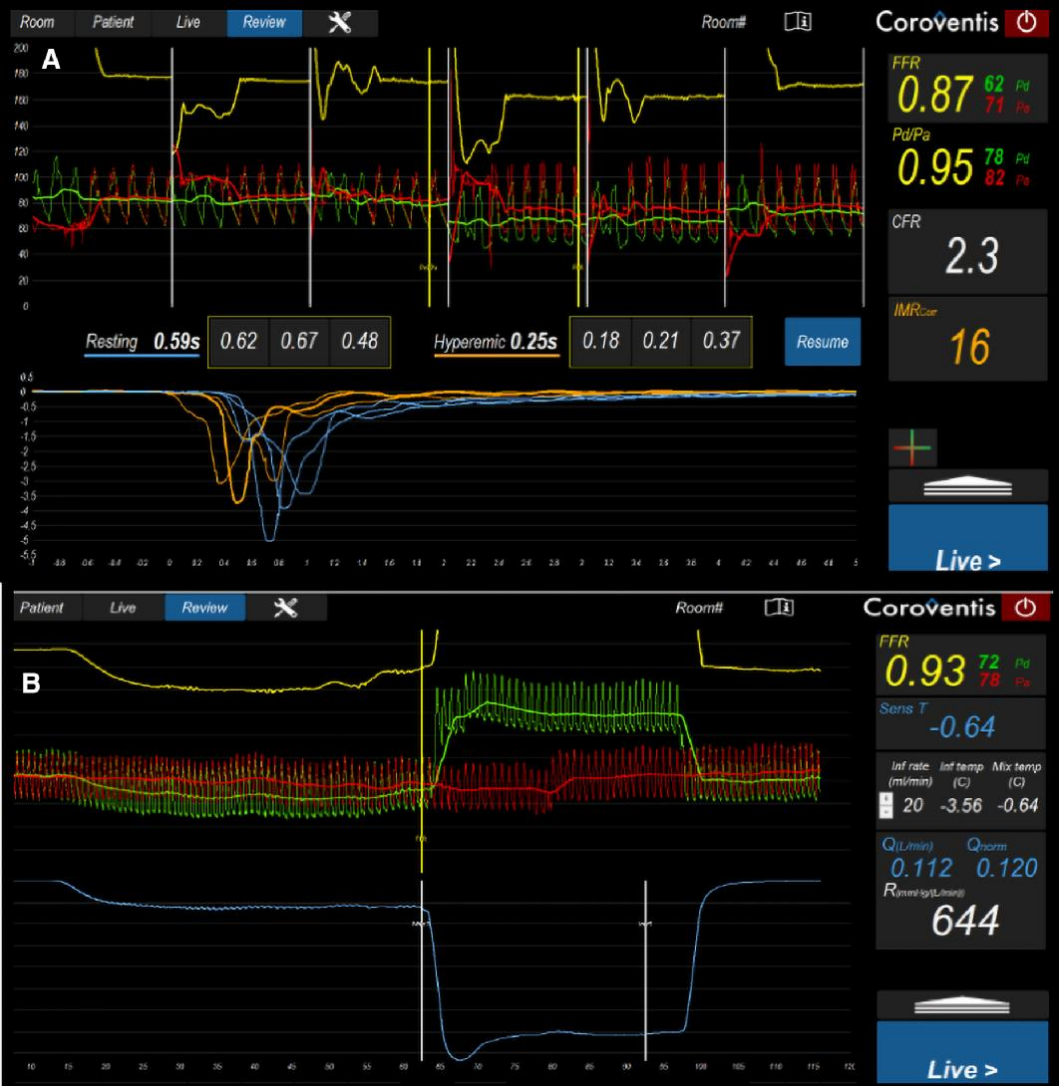
Invasive coronary function testing showing reduced vasodilatory reserve without structural microvascular disease. (*A*) Bolus thermodilution demonstrating reduced coronary flow reserve (CFR = 2.3) with normal microvascular resistance (IMR = 16). (*B*) Continuous thermodilution during saline infusion-induced hyperaemia confirming concordant findings (CFR = 2.2) with preserved microvascular resistance (*R* = 644 mmHg·s/L) and absolute flow (*Q* = 0.112 L/min), suggesting impaired vasodilatory reserve in the absence of increased microvascular resistance. Abbreviations: CFR, coronary flow reserve; IMR, index of microcirculatory resistance; Q, absolute coronary blood flow; R, resistance.

Given persistent symptoms, invasive coronary function testing using a dedicated infusion microcatheter system (RayFlow™, Hexacath) was performed in November 2024, showing no further epicardial lesion suitable for revascularization and a reduced coronary flow reserve (CFR 2.3 by bolus thermodilution and 2.2 by continuous thermodilution) with preserved microvascular resistance (IMR 16) (*Figure 3*). According to contemporary consensus definitions, a CFR <2.5 suggests impaired vasodilatory reserve, although the values observed here remain in a borderline range. In the absence of elevated IMR or acetylcholine-induced spasm, the findings were interpreted as predominantly functional impairment of vasodilatory capacity.

**Figure 4:**
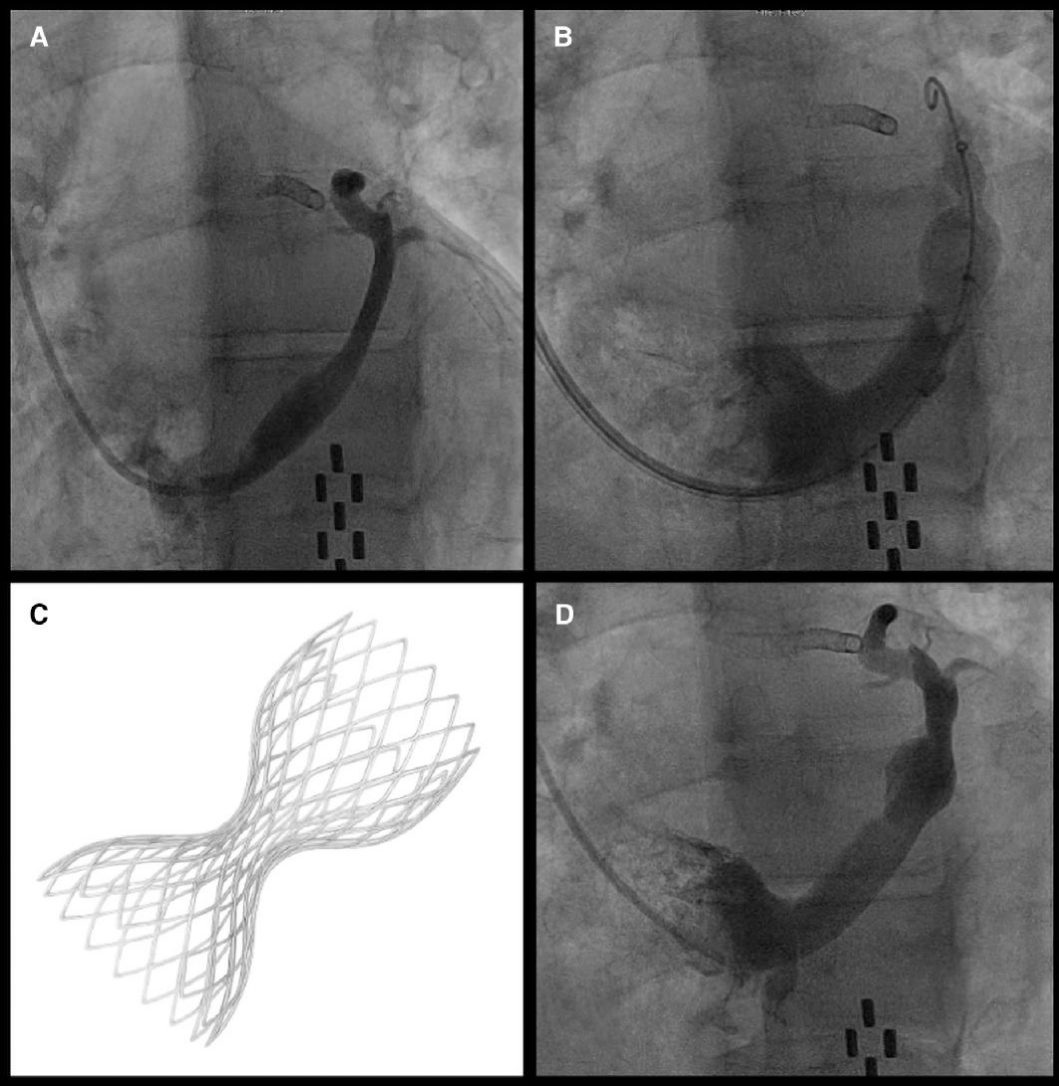
Coronary sinus reducer implantation. (*A*) Selective coronary sinus venography demonstrating a suitable landing zone for device implantation. (*B*) Fluoroscopic positioning and deployment of the Reducer in the mid-coronary sinus. (*C*) Schematic representation of the hourglass-shaped Reducer stent designed to create controlled narrowing and increase coronary venous pressure. (*D*) Post-implantation venogram confirming stable device position with preserved flow through the narrowed segment. Abbreviations: CSR, coronary sinus reducer.

Symptoms improved but persisted [Canadian Cardiovascular Society (CCS II)], despite maximally tolerated anti-anginal therapy including amlodipine 5 mg once daily, ranolazine 500 mg twice daily, metoprolol 25 mg once daily and lisinopril 2.5 mg once daily. Beta-blocker and angiotensin-converting enzyme inhibitor doses were limited by blood pressure and heart rate tolerance. After shared decision-making, CSR implantation was performed on 18 July 2025 as an off-label option for refractory angina without further revascularization targets (*Figure 4*).

At 3-month follow-up, the patient reported a clear subjective improvement in daily functioning and exercise tolerance following CSR implantation, with marked symptomatic improvement corresponding to CCS class I and resumption of unrestricted physical activity, including regular gym training. He remained clinically stable under dual antiplatelet therapy and optimized anti-anginal treatment, with no changes required given the sustained benefit.

## Discussion

This case illustrates how a contemporary, mechanism-oriented approach can help guide the evaluation and management of symptoms before and after PCI. The sequential use of angiography-derived physiology, invasive microcirculation assessment and, ultimately, CSR implantation reflects the broad spectrum of pathophysiological contributors that may coexist in a single patient.

Interpretation of invasive coronary function testing requires careful consideration of physiological thresholds. Contemporary consensus documents suggest that a CFR <2.5 indicates impaired vasodilatory reserve, whereas IMR ≥25 is typically used to define structural microvascular disease. In the present case, CFR values were modestly reduced (2.2–2.3) while IMR remained within normal range (16). This pattern is suggestive of a predominantly functional coronary microvascular dysfunction phenotype, characterized by impaired vasodilatory reserve in the presence of preserved microvascular resistance. Nevertheless, the borderline nature of CFR values warrants cautious interpretation, and alternative explanations such as diffuse epicardial disease or the impact of prior myocardial injury cannot be completely excluded.

The indication for CSR implantation in this case deserves careful consideration. Randomized evidence supporting CSR therapy, most notably the Coronary Sinus Reducer for Treatment of Refractory Angina (COSIRA) trial, primarily involved patients with obstructive coronary artery disease not amenable to further revascularization.^7^ In contrast, the present patient predominantly exhibited impaired vasodilatory reserve without demonstrable structural microvascular disease or epicardial spasm. Therefore, the use of CSR in this context should be regarded as extrapolative. The mechanistic rationale may relate to increased coronary venous pressure improving subendocardial perfusion and potentially modulating microvascular flow distribution. However, the possibility of placebo effects cannot be excluded, and the clinical improvement observed in this case was based primarily on symptom reporting rather than objective ischaemia reassessment. In addition, improvement following CSR implantation was assessed clinically based on symptom resolution and patient-reported functional capacity. No formal symptom questionnaire (such as the Seattle Angina Questionnaire) or repeat objective ischaemia testing was performed. Therefore, the observed benefit should be interpreted cautiously.

Although it is uncommon to deploy all three modalities in the same individual, this case underlines the importance of continuing the diagnostic pathway when symptoms persist despite apparently successful revascularization. Modern management increasingly recognizes the dynamic role of coronary microcirculation and, when no further epicardial target exists, the potential value of venous outflow modulation.

## Data Availability

The data underlying this article will be shared on reasonable request to the corresponding author.

## Lead author biography

Jérémie Buri graduated from the University of Lausanne in 2022 and completed two years of training in internal medicine followed by 6 months in intensive care. He is currently an MD candidate and cardiology resident at the University Hospital in Lausanne (CHUV), focusing on coronary physiology and ANOCA/INOCA patients.

## Notes

**Consent:** The authors confirm that written consent for submission and publication of this case report, including images and associated text, has been obtained from the patient, in line with COPE guidance.
